# Reproducibility and Potential Prognostic Value of Spatial Metabolic PET Metrics in Oral Tongue Squamous Cell Carcinoma

**DOI:** 10.3390/cancers18162637

**Published:** 2026-08-15

**Authors:** Soo Jin Kwon, Ie Ryung Yoo, Dong-Il Sun, Sang-Yeon Kim, Yeon-Sil Kim

**Affiliations:** 1 Division of Nuclear Medicine, Department of Radiology, Eunpyeong St. Mary’s Hospital, College of Medicine, The Catholic University of Korea, Seoul 03312, Republic of Korea; syung84@songeui.ac.kr; 2 Division of Nuclear Medicine, Department of Radiology, Seoul St. Mary’s Hospital, College of Medicine, The Catholic University of Korea, Seoul 06591, Republic of Korea; 3 Department of Otolaryngology–Head and Neck Surgery, Seoul St. Mary’s Hospital, College of Medicine, The Catholic University of Korea, Seoul 06591, Republic of Korea; hnsdi@catholic.ac.kr (D.-I.S.); ksyent@catholic.ac.kr (S.-Y.K.); 4 Department of Radiation Oncology, Seoul St. Mary’s Hospital, College of Medicine, The Catholic University of Korea, Seoul 06591, Republic of Korea; yeonkim7@catholic.ac.kr

**Keywords:** oral tongue cancer, squamous cell carcinoma, FDG PET/CT, radiomics, spatial metabolic metrics, prognosis

## Abstract

Background/Objectives: Conventional FDG PET parameters quantify metabolic intensity and volume but not the location of the metabolic hotspot, whose peripheral shift may reflect aggressiveness. In surgically treated oral tongue cancer (OTC), we evaluated two spatial PET metrics—the normalised distance from the hotspot to the tumour centroid (NHOCmax) and perimeter (NHOPmax)—in two parallel analyses: prognosis and measurement reproducibility. Methods: We retrospectively analysed 89 patients with preoperative FDG PET/CT. NHOCmax and NHOPmax were derived alongside conventional PET parameters. Associations with disease-free (DFS) and overall survival (OS) were assessed by uni and multivariable Cox models, and reproducibility was assessed across segmentation methods and by intra-reader repeatability. Results: Over a median 83.4/102.1-month follow-up (DFS/OS), 29 DFS and 25 OS events occurred. In the prognostic analysis, higher NHOCmax (OS HR 1.14, *p* = 0.032) and lower NHOPmax (DFS HR 0.65, *p* = 0.017; OS HR 0.65, *p* = 0.031) were associated with poorer survival upon univariable analysis. In the reproducibility analysis, both were robust to segmentation (intra-class correlation coefficient 0.94 for NHOCmax, 0.78 for NHOPmax); intra-reader repeatability was 0.69 and 0.94, respectively. Both metrics were less reproducible in low-volume tumours. Conclusions: NHOCmax and NHOPmax were associated with poorer survival, though not independently of stage and metabolic tumour volume, and were reproducible except in low-volume tumours; they warrant validation in larger cohorts.

## Simple Summary

Oral tongue cancer can behave aggressively even when detected early, so reliable preoperative markers of tumour behaviour are valuable. Conventional fluorodeoxyglucose (FDG) PET/CT parameters quantify the intensity and volume of tumour metabolism but not the spatial location of the most metabolically active region within the tumour. Emerging evidence suggests that a peripheral—rather than central—location of this metabolic hotspot may indicate more aggressive disease. In 89 patients with surgically treated oral tongue cancer, we measured the normalised distance of the metabolic hotspot from the tumour’s centre and from its edge on preoperative FDG PET/CT and examined how these measurements are related to survival and how reproducibility could be obtained. The spatial metrics were associated with survival in a consistent direction, were reproducible except in small tumours, and conveyed information not captured by conventional PET parameters, supporting further study of both their prognostic value and their measurement reliability.

## 1. Introduction

Mathematical biology has emerged as a pivotal framework for deciphering the growth dynamics of malignant tumours in humans. Across various cancer types, tumour growth was observed to follow a superlinear scaling law in fluorine-18 fluorodeoxyglucose (FDG) positron emission tomography/computed tomography (PET/CT) and other metabolic tracer studies, in which metabolic expansion becomes increasingly explosive as the lesion enlarges [1]. In such a relationship, the total metabolic activity of a tumour does not increase in proportion to its volume but grows faster than proportionally—that is, with a power-law exponent greater than one—so that metabolic activity per unit volume rises as the tumour enlarges. Building on this framework, Jiménez-Sánchez et al. reported that as a tumour expands, the most active proliferative clones migrate from the hypoxic core toward the nutrient-rich periphery [2]. To quantify this phenomenon, spatial metrics, such as the normalised distance from the hotspot to the tumour centroid (NHOC) and the normalised distance to the tumour perimeter (NHOP), were developed, with the peripheral localisation of metabolic activity proposed as an indicator of unfavourable clinical outcomes.

While the superlinear metabolic scaling law has been observed in various malignancies, including head and neck cancer [1], the clinical validation of these spatial metrics has predominantly been explored in lung [3,4,5,6,7] and breast cancers [8]. Although a recent study investigated these metrics in head and neck squamous cell carcinoma, it was limited to patients treated with chemoradiation [9]. Consequently, their prognostic value in surgically treated oral tongue cancer (OTC) remains largely unexplored. Cancer of the oral cavity was diagnosed in an estimated 317,500 people worldwide in 2022 [10]. The tongue was the most common subsite, accounting for 52.8% of the oral squamous cell carcinomas recorded in the Japanese national cancer registry between 2016 and 2019 [11]. Tongue cancer incidence has increased in Korea, where the annual percent change between 1999 and 2017 was 7.7% at ages 20 to 39 years and 2.7% at 60 years or older [12]. OTC is characterised by a high propensity for local invasion and cervical lymph node metastasis, even in early stages [13]—behaviours that may be intrinsically linked to the peripheral migration of aggressive clones. FDG PET/CT and its conventional parameters have shown good diagnostic and prognostic performance and cost-effectiveness in patients with OTC [14,15,16]. However, FDG PET/CT still falls short in predicting occult nodal metastases and correlates variably with pathological findings [17]. Therefore, there is a critical unmet need for non-invasive preoperative imaging biomarkers that can predict aggressive tumour biology and support preoperative risk stratification in OTC.

We hypothesised that PET-derived spatial metrics could serve as non-invasive indicators of aggressive phenotypes and provide additional prognostic value beyond that of conventional PET parameters. This study therefore addressed two distinct endpoints in parallel. First, we evaluated the associations of NHOCmax and NHOPmax with clinicopathological findings and survival in surgically treated OTC. Second, we evaluated whether the two metrics can be measured reproducibly across segmentation methods, on repeat segmentation by the same reader, and across tumour volumes.

## 2. Materials and Methods

### 2.1. Patients

**Figure 1:**
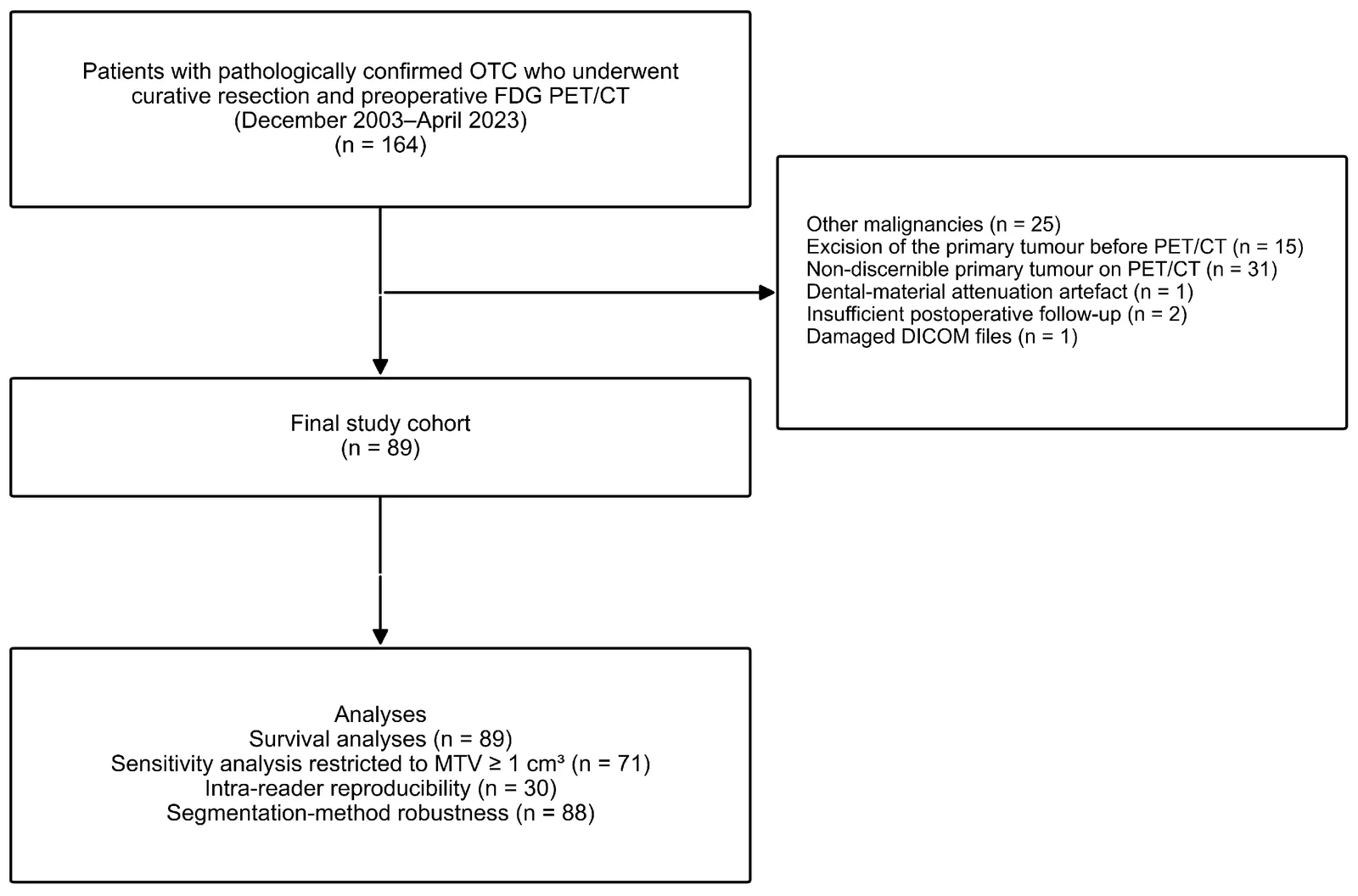
Flow chart of patient selection. OTC = oral tongue cancer; PET/CT = positron emission tomography/computed tomography; FDG = fluorodeoxyglucose; MTV = metabolic tumour volume.

This retrospective single-centre study included patients with pathologically confirmed OTC who underwent curative resection of the primary tumour and preoperative baseline FDG PET/CT at our institution between December 2003 and April 2023. Patients with other malignancies, those who received neoadjuvant chemotherapy, those who underwent primary tumour excision before PET/CT, and those with non-discernible primary tumours on PET/CT were excluded from the study. Patients were also excluded for intense FDG uptake related to a dental material attenuation artefact, insufficient postoperative follow-up, or damaged DICOM files. Dental material attenuation artefacts were assessed by two nuclear medicine physicians who reviewed the attenuation-corrected and non-attenuation-corrected PET images together (Figure 1). Clinicopathological variables including tumour size, depth of invasion (DOI), perineural invasion (PNI), lymphovascular invasion (LVI), lymph node metastasis, resection margin, adjuvant treatment, and survival outcomes were retrieved from electronic medical records. All tumours were staged according to the 8th edition of the AJCC staging system, with cases from earlier periods restaged accordingly. The dates of surgery, recurrence, death, and last follow-up were recorded. Tumour recurrence was confirmed using biopsy or serial imaging. This study was approved by the Institutional Review Board (approval number: KC25RISI0787), and the requirement for informed consent was waived due to the retrospective nature of the study.

### 2.2. Acquisition of F-18 FDG PET/CT

Prior to the scans, all patients fasted for at least 6 h, and their blood glucose levels were confirmed to be <200 mg/dL. 18F-FDG was administered intravenously at a dosage of 3.7–5.5 MBq/kg. PET/CT imaging was performed approximately 60 min after radiotracer injection using one of three scanner systems: Siemens Biograph DUO, Siemens Biograph TruePoint (Siemens Healthcare, Erlangen, Germany), and GE Discovery 710 (GE HealthCare, Chicago, IL, USA) scanners.

CT scans for attenuation correction and anatomical localisation were acquired. Following CT acquisition, attenuation-corrected PET images were reconstructed using scanner-specific reconstruction parameters (Table S1).

### 2.3. Tumour Segmentation and Image Analysis

All image processing, tumour segmentation, and parameter extraction were performed using LIFEx software (version 25.06.1) [18]. First, the anonymised PET Digital Imaging and Communications in Medicine (DICOM) images were resampled to an isotropic voxel size of 4 × 4 × 4 mm using the default interpolation implemented in LIFEx. Native voxel dimensions differed among the three scanners and were anisotropic in each of them (in-plane, 3.65–5.31 mm; slice thickness, 3.27–5.00 mm; Table S1). Because NHOCmax and NHOPmax are three-dimensional Euclidean distances derived from voxel coordinates, an isotropic grid ensures that a given distance carries the same physical meaning in every direction, and a grid common to all studies ensures that the values are comparable across scanners. A 4 mm isotropic voxel was chosen because it lies within the range of the native dimensions so that no scanner underwent pronounced up or downsampling. For the primary (reference) segmentation, a three-dimensional volume of interest was manually delineated for each primary tongue tumour, and voxels within it were retained by applying both an absolute standardised uptake value (SUV) threshold of 2.5 and a relative threshold of 41% of SUVmax—a percentage-of-SUVmax isocontour recommended by the EANM procedure guidelines for tumour imaging [19]—with a voxel included only when it satisfied both criteria. The boundaries and shapes of the volumes of interest were confirmed by consensus between two nuclear medicine physicians (with 9 and 20 years of experience in PET/CT interpretation); any discrepancies were resolved by joint re-review until consensus was reached. This manual reference segmentation, which accounted for peritumoural physiologic activity, was used for primary and survival analyses.

**Figure 2:**
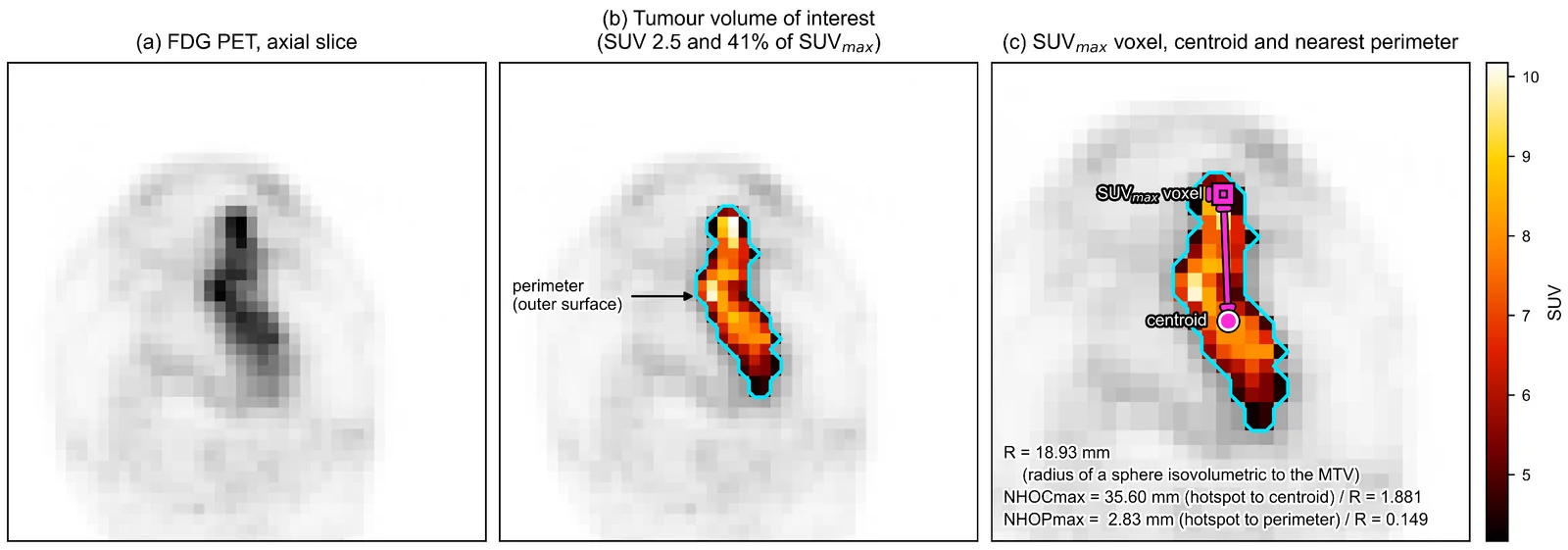
Measurement of NHOCmax and NHOPmax in an example case. (**a**) Axial FDG PET image. (**b**) The delineated tumour volume of interest (SUV 2.5 combined with 41% of SUVmax); the outline is its perimeter, that is, the outer surface of the delineated volume. (**c**) Two distances are measured from the SUVmax voxel (square): one to the centroid (circle), shown by the pink line, and one to the nearest perimeter. Each was then normalised by R, the radius of a sphere isovolumetric to the MTV, to give NHOCmax and NHOPmax, respectively. The hotspot lies close to the surface, giving a low NHOPmax (0.149) and a high NHOCmax (1.881). Abbreviations: FDG = fluorodeoxyglucose; PET = positron emission tomography; SUV = standardised uptake value; SUVmax = maximum standardised uptake value; MTV = metabolic tumour volume; NHOCmax = normalised distance from the hotspot to the centroid; NHOPmax = normalised distance from the hotspot to the perimeter.

Conventional metabolic parameters (SUVmax, SUVmean, MTV, and TLG) and spatial PET metrics (NHOCmax and NHOPmax) were extracted automatically in LIFEx, as previously described [4]. For both spatial metrics, the hotspot was the single voxel of maximum uptake (the SUVmax voxel). NHOCmax was defined as the Euclidean distance between the SUVmax voxel and the centroid of the segmented volume. NHOPmax was defined as the shortest three-dimensional distance from the SUVmax voxel to the nearest voxel on the outer surface (referred to as the “perimeter” in the LIFEx software and the metric nomenclature) of the segmented volume (Figure 2). Both distances were normalised by R, the radius of a sphere isovolumetric to the MTV, calculated as R = (3 × MTV/4π) ^(1/3)^. SUVpeak-based spatial metrics (NHOCpeak and NHOPpeak) were defined identically but used the SUVpeak voxel—the centre of a 1 cm^3^ spherical volume positioned to maximise the mean uptake—as the hotspot instead of the SUVmax voxel. Because a 1 cm^3^ peak sphere can be placed only in sufficiently large tumours, these metrics were computable in a subset of tumours and were used only in the reproducibility analysis, not in the survival analyses.

To assess segmentation-method robustness, six alternative segmentations were additionally generated for each tumour using an in-house Python (version 3.14.4) pipeline based on an optimal-surface segmentation method [20], implemented in 3D Slicer (version 5.10.0) [21], each applying a different threshold from among the fixed-SUV and percentage-of-SUVmax methods, as previously described [22]: absolute SUV thresholds of 2.5 and 3.0; relative thresholds of 40%, 41%, and 50% of SUVmax; and a combined rule (the greater of SUV 2.5 or 41% of SUVmax). These alternative segmentations were generated automatically and were not manually adjusted for adjacent physiologic uptake, so as to isolate the effect of varying the segmentation method. One tumour was excluded because its SUVmax was below 3.0, so the fixed SUV = 3.0 segmentation produced an empty volume that precluded feature extraction.

### 2.4. Reproducibility and Robustness Analyses

The reproducibility of NHOCmax and NHOPmax was evaluated in two complementary analyses, with the results stratified by the reference-segmentation MTV (<1, 1–2, 2–5, and ≥5 cm^3^).

First, intra-reader reproducibility was evaluated. A single reader manually re-segmented a balanced subset of 30 tumours in LIFEx (spanning all MTV strata) after a washout interval, and the agreement between the original and repeated segmentations was quantified by the intra-class correlation coefficient (ICC).

Second, segmentation-method robustness was quantified by re-extracting NHOCmax and NHOPmax from each of the six alternative segmentations described above and computing their agreement across the six segmentations using the ICC; this analysis was based on the 88 tumours with complete values across all six segmentations. The SUVpeak-based spatial metrics (NHOCpeak and NHOPpeak) were evaluated in the same manner in the subset of tumours in which a 1 cm^3^ peak sphere could be placed across all six segmentations (Table S2).

### 2.5. Statistical Analyses

Statistical analyses were conducted using R software (version 4.5.1) [23]. All tests were two-sided, and a *p*-value < 0.05 was considered statistically significant. Descriptive statistics are presented as means with standard deviations for continuous variables, frequencies for categorical variables, and medians with inter-quartile ranges for the PET parameters, which were non-normally distributed. All survival analyses were performed on the full cohort (*n* = 89) using complete cases; a sensitivity analysis restricted to MTV ≥ 1 cm^3^ (*n* = 71) was additionally performed because the SUVmax-based spatial metrics are less reliable in small lesions.

Correlations among the PET-derived parameters (SUVmax, SUVmean, TLG, MTV, NHOCmax, and NHOPmax) were assessed using Spearman’s rank correlation, with *p*-values adjusted for multiple comparisons using the Benjamini–Hochberg false discovery rate (FDR). Differences in PET parameters across clinicopathological subgroups (nodal metastasis, LVI, and PNI) were evaluated using exact Mann–Whitney U tests, and likewise with Benjamini–Hochberg FDR correction.

Disease-free survival (DFS) was defined as the time from surgery to recurrence or death from any cause, and overall survival (OS) was the time to death from any cause; event-free patients were censored at their last follow-up, and median follow-up was estimated by the reverse Kaplan–Meier method. Univariable Cox proportional-hazards models were fitted for all clinicopathological and PET parameters for descriptive reporting. For the multivariable models, given the limited number of events, pre-specified variables were used: AJCC stage (I–III vs. IV), MTV, and each spatial metric (NHOCmax or NHOPmax), modelled continuously, was entered in separate models. Incremental prognostic value over stage and MTV was assessed by nested likelihood-ratio tests, and model discrimination was assessed by Harrell’s C-index, with optimism-corrected estimates and 95% confidence intervals obtained by the location-shifted bootstrap method (B = 1000) [24]. The proportional-hazards assumption was checked with scaled Schoenfeld residuals, and the log-linearity of continuous covariates was checked by comparing the linear specification with a restricted cubic-spline term (likelihood-ratio test). Unlike the correlation and subgroup analyses, the survival analyses were not adjusted for multiple comparisons; their *p*-values are interpreted descriptively.

Kaplan–Meier survival curves were generated and compared using log-rank tests, with the spatial metrics dichotomised both at cut-offs derived from maximally selected rank statistics (maxstat R package) [25] and at their median value.

Inter-segmentation and intra-reader agreement were summarised using the two-way random-effects, single-measure ICC for absolute agreement, reported with 95% confidence intervals and classified as poor (<0.50), moderate (0.50–0.75), good (0.75–0.90), or excellent (>0.90) [26].

## 3. Results

### 3.1. Patient Characteristics

Of 164 patients with pathologically confirmed OTC who underwent preoperative PET/CT during the study period, 75 were excluded (25 for other malignancies, 15 for excision of the primary tumour before PET/CT, 31 for a non-discernible primary tumour on PET/CT, one for intense FDG uptake related to dental material attenuation artefacts, two for insufficient postoperative follow-up, and one for damaged DICOM files), leaving 89 patients for the final analysis. The baseline characteristics of the patients are summarised in Table 1. The mean age was 52 years, and 58% of the patients were male. The positivity rates for LVI and PNI were 28% and 42%, respectively. According to the American Joint Committee on Cancer (AJCC) 8th edition staging system, the cohort comprised stage I (*n* = 21, 24%), stage II (*n* = 20, 22%), stage III (*n* = 26, 29%), and stage IV (*n* = 22, 25%) diseases. One patient with a missing DOI measurement was assigned to stage IVB based on N3 disease, so the missing value did not affect the stage assignment. One-third of the cohort (34%) received adjuvant postoperative therapy (radiation, chemoradiation, or chemotherapy).

### 3.2. PET Parameters: Distribution and Clinicopathological Correlations

**Figure 3:**
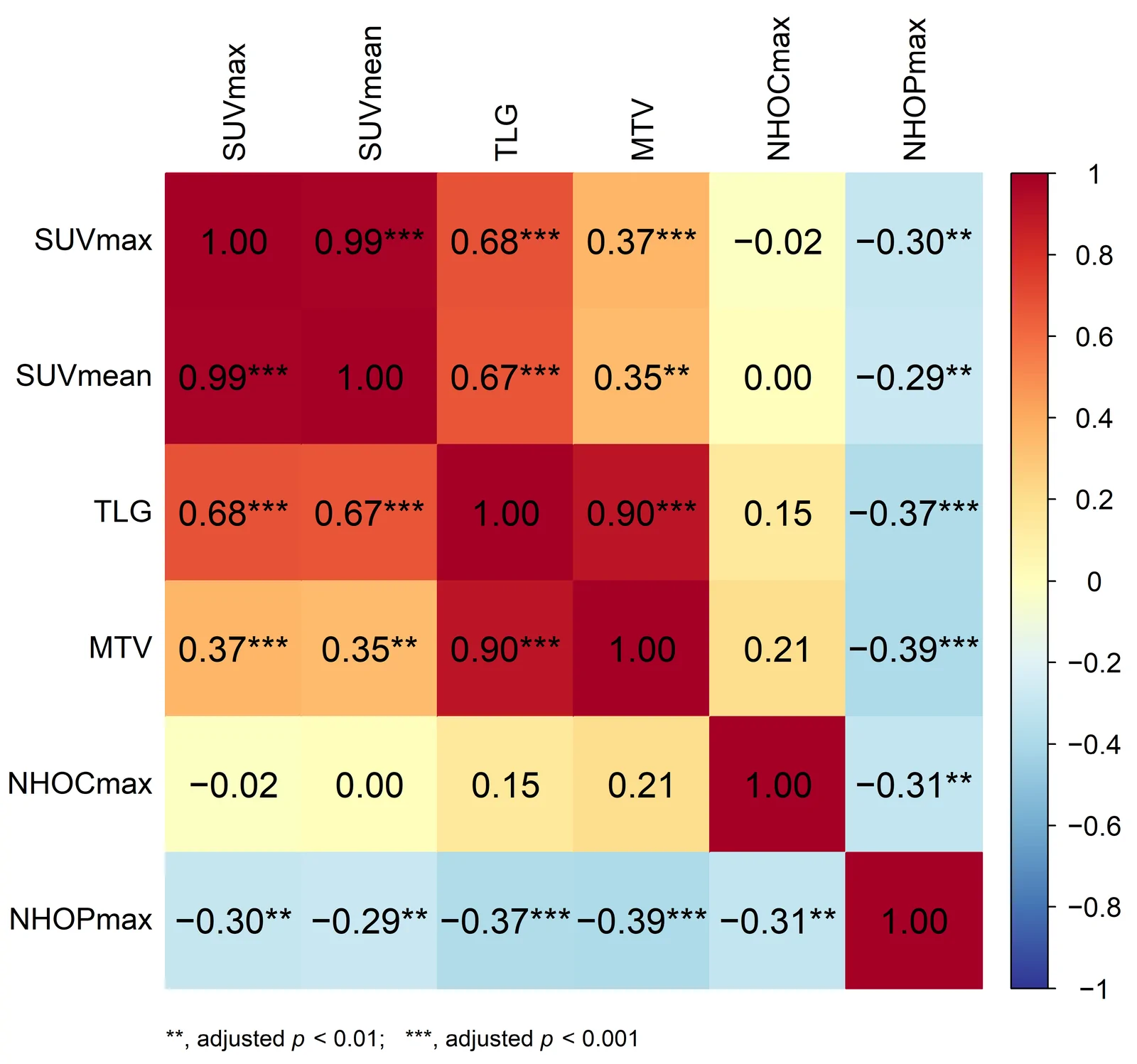
Correlogram of Spearman rank correlation coefficients (ρ) among the PET parameters. *p*-values were adjusted for multiple comparisons using the Benjamini–Hochberg false discovery rate, and asterisks denote significance (**, adjusted *p* < 0.01; ***, adjusted *p* < 0.001). Abbreviations: SUVmax = maximum standardised uptake value; SUVmean = mean standardised uptake value; TLG = total lesion glycolysis; MTV = metabolic tumour volume; NHOCmax = normalised distance from the hotspot to the centroid; NHOPmax = normalised distance from the hotspot to the perimeter.

Table 2 shows the distribution of PET parameters in the primary cohort (*n* = 89). The median values were 0.39 (IQR 0.26–0.60) for NHOCmax and 0.43 (IQR 0.34–0.50) for NHOPmax. Figure 3 shows the correlations among the PET parameters. NHOCmax showed no significant correlation with any conventional parameter (SUVmax ρ = −0.02, *p* = 0.950; SUVmean ρ = 0.00, *p* = 0.970; MTV ρ = 0.21, *p* = 0.060; TLG ρ = 0.15, *p* = 0.190). NHOPmax showed inverse correlations with SUVmax (ρ = −0.30, *p* = 0.007), SUVmean (ρ = −0.29, *p* = 0.009), MTV (ρ = −0.39, *p* < 0.001), and TLG (ρ = −0.37, *p* < 0.001). NHOCmax and NHOPmax were inversely correlated with each other (ρ = −0.31, *p* = 0.006).

**Figure 4:**
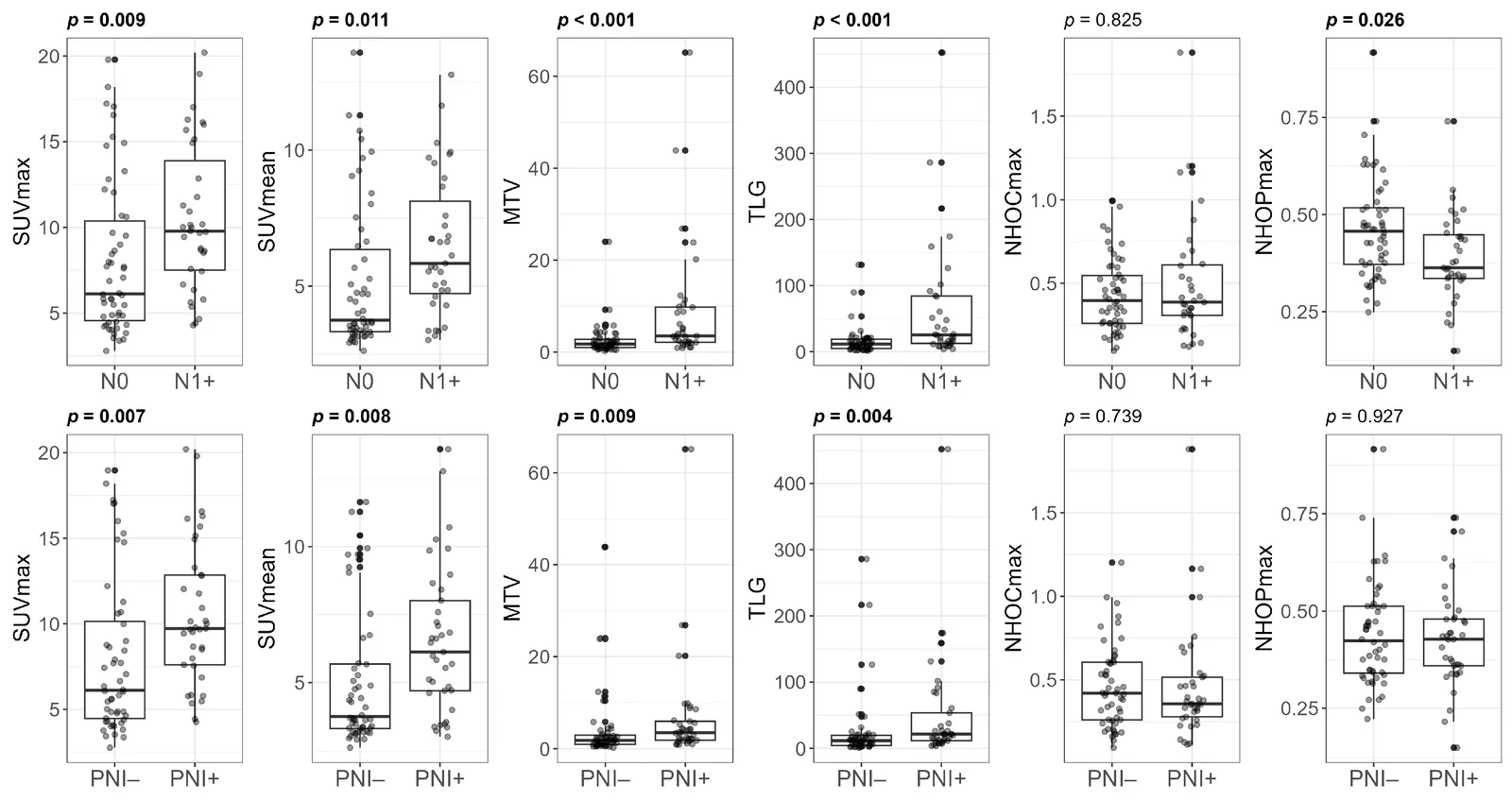
Boxplots comparing SUVmax, SUVmean, MTV, TLG, NHOCmax, and NHOPmax among the subgroups of lymph node positivity (N0 vs. N1+) and perineural invasion (PNI− vs. PNI+). Each panel title shows the Benjamini–Hochberg-adjusted Mann–Whitney *p*-value; significant comparisons (adjusted *p* < 0.05) are shown in bold. Abbreviations: SUVmax = maximum standardised uptake value; SUVmean = mean standardised uptake value; MTV = metabolic tumour volume; TLG = total lesion glycolysis; NHOCmax = normalised distance from the hotspot to the centroid; NHOPmax = normalised distance from the hotspot to the perimeter.

We compared PET parameters among the clinical subgroups (nodal metastasis, LVI, and PNI) (Figure 4). Patients with lymph node metastasis (N1+) had significantly higher SUVmax, SUVmean, MTV, and TLG than those without nodal metastasis (N0) (all *p* < 0.05). NHOPmax was significantly lower in the N1+ group (*p* = 0.026), whereas NHOCmax showed no significant difference. The PNI-positive group had significantly higher SUVmax, SUVmean, MTV, and TLG values (all *p* < 0.05), whereas NHOCmax and NHOPmax did not differ significantly according to PNI status. No significant differences were observed in any of the PET parameters regarding LVI status (all *p* > 0.05).

### 3.3. Survival Analysis

The median follow-up was 83.4 months for DFS and 102.1 months for OS. DFS events (recurrence or death) occurred in twenty-nine patients (32.6%): tumour recurrence in twenty patients (22.5%) and death without prior documented recurrence in nine patients (10.1%). Locoregional recurrence and distant metastasis occurred in nineteen and four patients, respectively. A total of 25 deaths (28.1%) occurred during the study period.

Upon univariable Cox analysis (Table 3), tumour size, depth of invasion, PNI, and advanced AJCC stage (IV vs. I–III) were associated with both DFS and OS. Older age was associated with shorter OS. Among the PET parameters, higher TLG and MTV were associated with poorer DFS and OS, whereas SUVmax and SUVmean were not. Adjuvant treatment was not associated with either survival outcome (log-rank *p* = 0.670 for DFS and 0.938 for OS).

For the spatial metrics (per 0.1-unit increment), a higher NHOCmax was associated with a higher event rate for OS (HR 1.14, 95% CI 1.01–1.29, *p* = 0.032); a similar trend was observed for DFS, although it did not reach statistical significance (HR 1.11, 95% CI 0.99–1.23, *p* = 0.063). A lower NHOPmax was associated with a higher event rate for both survival outcomes (DFS HR 0.65, 0.45–0.92, *p* = 0.017; OS HR 0.65, 0.44–0.96, *p* = 0.031).

In the multivariable models (Table 4), NHOCmax and NHOPmax were each entered separately, together with AJCC stage and MTV. After adjustment, a higher NHOCmax and a lower NHOPmax each showed a trend toward poorer survival, but neither reached statistical significance (NHOCmax DFS HR 1.08, 95% CI 0.96–1.20, *p* = 0.199; OS HR 1.09, 0.96–1.23, *p* = 0.182; NHOPmax DFS HR 0.75, 0.53–1.06, *p* = 0.101; OS HR 0.81, 0.56–1.18, *p* = 0.273). The proportional-hazards assumption was satisfied, and no departure from log-linearity was detected for any continuous covariate in all the multivariable models (all global Schoenfeld and log-linearity *p* > 0.05).

In the sensitivity analysis restricted to MTV ≥ 1 cm^3^ (*n* = 71), both spatial metrics retained the same direction of association upon univariable Cox analysis. A higher NHOCmax remained associated with worse OS (HR 1.15, 95% CI 1.02–1.30, *p* = 0.025), whereas the remaining univariable associations did not reach statistical significance. Neither metric remained significant after adjustment for AJCC stage and MTV (Table S3).

Upon nested likelihood-ratio testing, adding NHOCmax or NHOPmax to a base model of AJCC stage and MTV did not reach significance (NHOCmax DFS *p* = 0.230, OS *p* = 0.210; NHOPmax DFS *p* = 0.088, OS *p* = 0.260). The optimism-corrected C-index of the base model (AJCC stage and MTV) was 0.642 (95% CI 0.531–0.750) for DFS and 0.695 (0.569–0.816) for OS; adding a spatial metric yielded 0.656 (0.537–0.773; NHOCmax) and 0.679 (0.571–0.781; NHOPmax) for DFS, and 0.703 (0.579–0.827; NHOCmax) and 0.700 (0.585–0.813; NHOPmax) for OS, with widely overlapping confidence intervals.

**Figure 5:**
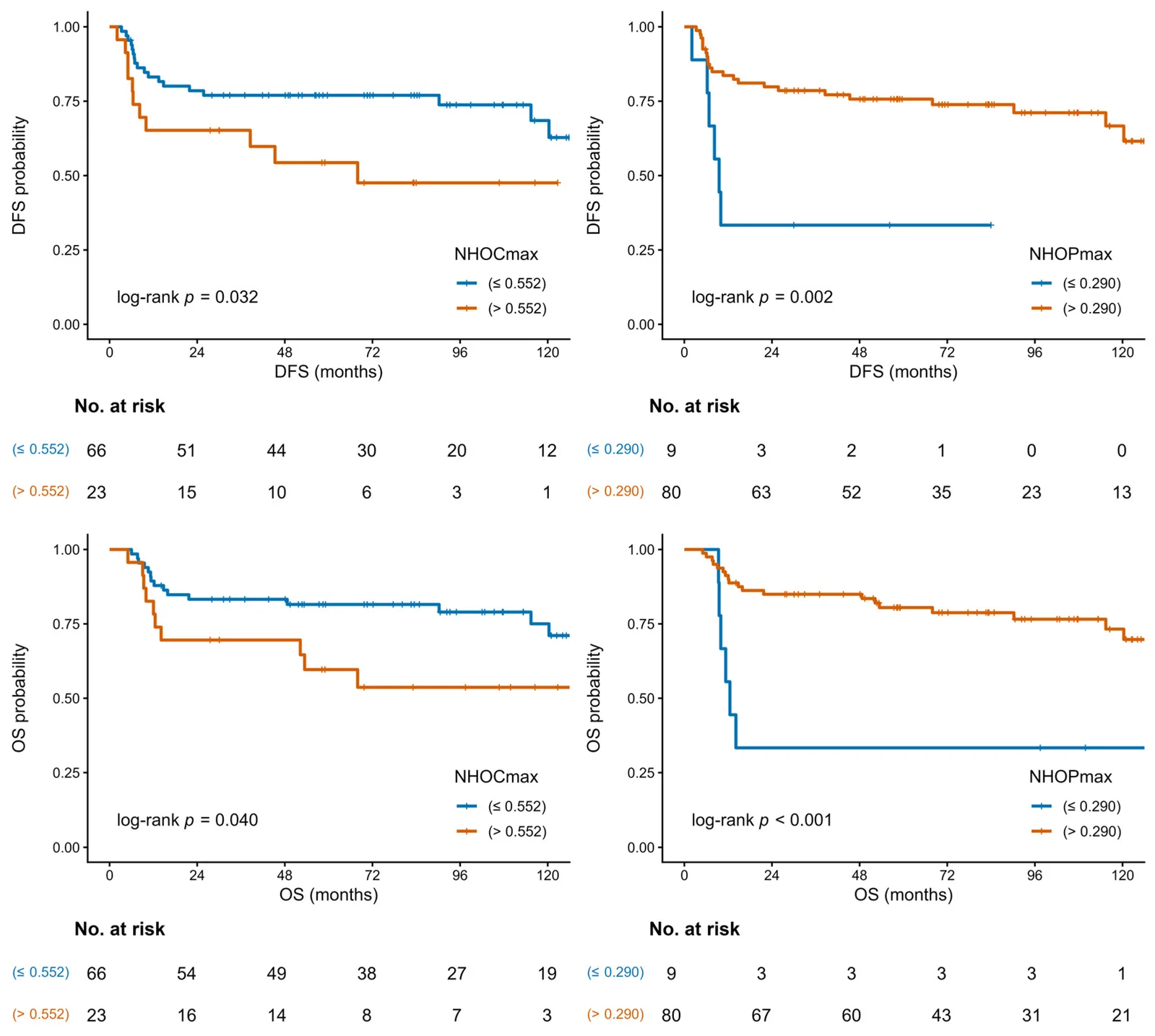
Kaplan–Meier curves for NHOCmax (first column) and NHOPmax (second column), with DFS in the top row and OS in the bottom row. The cutoff value for each metric was calculated using the maximally selected rank statistics and is indicated in the panel legend (low: ≤cutoff; high: >cutoff). The log-rank *p*-value is shown in each graph. Abbreviations: DFS = disease-free survival; OS = overall survival; PET = positron emission tomography; SUVmax = maximum standardised uptake value; MTV = metabolic tumour volume; TLG = total lesion glycolysis; NHOCmax = normalised distance from the hotspot to the centroid; NHOPmax = normalised distance from the hotspot to the perimeter.

NHOCmax and NHOPmax were dichotomised at cutoffs derived from maximally selected rank statistics (Figure 5). A higher NHOCmax (cutoff 0.552; *n* = 23 vs. 66) was associated with shorter DFS (log-rank *p* = 0.032) and OS (*p* = 0.040). A lower NHOPmax (cutoff 0.290; *n* = 9 vs. 80) was associated with shorter DFS (*p* = 0.002) and OS (*p* < 0.001). When the two spatial metrics were instead dichotomised at their median values, the survival differences did not reach statistical significance (Figure S1). Kaplan–Meier curves for the conventional PET parameters are provided in Figure S2.

### 3.4. Reproducibility of the Spatial Metrics

For intra-reader repeatability (repeated manual segmentation, *n* = 30), NHOPmax showed excellent agreement across all volume strata (ICC 0.94). NHOCmax agreement was volume-dependent: low below 1 cm^3^ (ICC 0.15), moderate at 1–2 cm^3^ (ICC 0.52), good to excellent at 2 cm^3^ or above (ICC 0.85–0.92), and moderate overall (ICC 0.69) (Table 5).

Across six automated segmentation methods (*n* = 88), NHOCmax showed excellent agreement (ICC 0.94) and NHOPmax showed good agreement (ICC 0.78), the latter declining in low-volume tumours (ICC 0.55) (Table 6).

The SUVpeak-based metrics could be computed across all six segmentations in only 22 tumours; in this subset, NHOCpeak showed excellent agreement (ICC 0.93) and NHOPpeak showed good agreement (ICC 0.88) (Table S2).

## 4. Discussion

In this study of surgically treated OTC, higher NHOCmax was associated with shorter OS and lower NHOPmax was associated with shorter DFS and OS upon univariable analysis; after adjustment for AJCC stage and MTV, both associations persisted in direction without reaching statistical significance. Higher NHOCmax and lower NHOPmax describe the same geometry: a hotspot lying further from the centre and closer to the edge, the direction predicted by the evolutionary model behind the metrics [2]. In the reproducibility analysis, both were measured consistently across segmentation methods and upon intra-reader re-segmentation, with agreement falling only in low-volume tumours.

The prognostic associations of the spatial metrics were consistent with reports in other solid tumours. In resectable NSCLC, the normalised SUVmax-to-perimeter distance (nSPD), a two-dimensional spatial metric analogous to NHOPmax, was identified as a strong independent predictor of early recurrence and short-term mortality, with lower nSPD values indicating poorer outcomes [3]. A machine-learning model combining NHOC parameters showed high performance in predicting lymph node metastasis, linking elevated NHOC values to tumour aggressiveness [6]. Our study extended these observations to surgically treated OTC, which, to our knowledge, had not previously been examined with these spatial metrics. The only prior head and neck report was restricted to patients treated with chemoradiation [9].

Both MTV and TLG were associated with survival in our cohort (Table 3), consistent with their established prognostic role in head and neck cancer [27,28]. These volumetric parameters, however, summarise the overall metabolic burden rather than the location of the metabolic hotspot within the tumour. Both NHOCmax and NHOPmax were only weakly related to the intensity- and volume-based PET parameters: NHOCmax was essentially uncorrelated with metabolic intensity and volume, whereas NHOPmax showed only weak inverse correlations with SUVmax, SUVmean, MTV, and TLG (Figure 3). A possible pathological correlate of this spatial information emerged in the subgroup analysis: NHOPmax was significantly lower in patients with nodal metastasis, whereas NHOCmax did not differ (Figure 4). A lower NHOPmax suggests a metabolic hotspot lying closer to the tumour boundary, which may place the most active subclones nearer the rich lymphatic network of the tongue and thereby favour nodal spread.

NHOCmax and NHOPmax were derived from a mathematical model of tumour evolution rather than from histology [2]. The invasive front has nonetheless long been proposed as a region informative for prognostication in oral carcinoma [29], and its histological features carry prognostic weight in oral tongue cancer: a risk model combining the modified worst pattern of invasion with tumour budding (clusters of fewer than five cancer cells at the invasive front) predicted disease-specific and disease-free survival [30]. Tumour budding has likewise been associated with nodal metastasis in oral squamous cell carcinoma [31]. These histological features are assessed at a scale finer than the 4 mm voxels used here, and to our knowledge, neither NHOCmax nor NHOPmax have been compared with them in the same tumours; the parallel is conceptual. Among the invasion markers available, neither NHOCmax nor NHOPmax differed by PNI or LVI status (Figure 4), although spatial metrics were associated with lymphovascular invasion in NSCLC [5].

Reproducibility depended on tumour volume and differed between the two metrics. Unlike metabolic tumour volume, which is defined directly by the segmentation threshold, NHOCmax depends on the centroid of the entire delineated volume and was robust to the choice of threshold. NHOPmax, measured to the nearest point on the boundary, was more sensitive to it. Agreement was good to excellent in tumours larger than 2 cm^3^ but fell in low-volume tumours. This is likely due to the difficulty of localising the maximum-uptake voxel in small lesions, where partial-volume effects and the limited spatial resolution of PET are most pronounced. However, these low-volume estimates were based on few tumours per stratum and had wide confidence intervals. Inter-reader agreement could not be assessed; the segmentations were confirmed by consensus with a more experienced reader.

The SUVpeak-based metrics (NHOCpeak and NHOPpeak) were not included in the survival analysis because SUVpeak is computed within a fixed 1 cm^3^ sphere that small tumours cannot accommodate. These metrics could be derived in only 37 of the 89 tumours, in which 15 DFS and 14 OS events occurred—too few for a reliable survival analysis. The oral tongue is a small organ, and surgically treated OTC is typically detected at a limited size. Even when volume exceeded 1 cm^3^, a superficially spreading shape (broad in extent but shallow in depth) could preclude placement of the sphere. In the smaller subset of 22 tumours in which they could be measured across all six segmentations, the peak metrics were reproducible overall (NHOCpeak ICC 0.93, NHOPpeak ICC 0.88; Table S2).

In this cohort, we could not demonstrate superiority over the conventional volumetric parameters or incremental prognostic value beyond AJCC stage and MTV. NHOCmax and NHOPmax instead may capture information distinct from intensity and volume, and each was reproducible against a different source of variation—NHOCmax when the segmentation method changed and NHOPmax when the same reader re-segmented (ICC 0.94 for both); in NSCLC, such spatial descriptors have been combined with radiomic features into composite signatures rather than used alone [6,32]. A similar composite, combining these spatial metrics with the conventional volumetric parameters, could be examined in OTC. Three questions remain for future studies: Whether the metrics track the histology of the invasive front would require pairing PET with a dedicated assessment of tumour budding and the worst pattern of invasion. Whether they add prognostic information beyond stage and MTV would require approximately 60–200 events for the adjusted hazard ratios observed in this cohort (Table 4), assuming a two-sided α of 0.05 and 80% power [33]; because those estimates are themselves uncertain, the true requirement may be larger. Whether they generalise to other head and neck subsites, which remains to be examined. We will extend this work to subsites such as the hypopharynx, where physiological uptake in the surrounding tissue is less pronounced.

Our study has several limitations. First, this was a retrospective analysis of a single-institution cohort limited to surgically treated patients, which constrains generalisability to other treatment settings and populations.

Second, the cohort was modest in size with few events, and no external validation cohort was available; discrimination was therefore assessed only internally (optimism-corrected by bootstrapping), and calibration was not formally evaluated. The multivariable models were consequently confined to a small set of pre-specified covariates (AJCC stage and MTV); established prognosticators significant upon univariable analysis—tumour size, DOI, and PNI—could not be included, so residual confounding cannot be excluded. The dichotomised survival analyses should likewise be regarded as illustrative, their cut-offs—derived within the same cohort—requiring validation in independent cohorts. The sensitivity analysis restricted to MTV ≥ 1 cm^3^ had still fewer events (24 for DFS and 21 for OS).

Third, imaging spanned three PET/CT scanners over nearly two decades. Although PET images were resampled to a common voxel size, no dedicated cross-scanner harmonisation (e.g., ComBat) was performed, and the per-scanner numbers were too small for scanner-stratified survival analysis. SUVmax is an intensity-based parameter that varies with reconstruction and scanner generation [34]. The spatial metrics, by contrast, are based on the location of the maximum-uptake voxel, and although this position may shift with image smoothing, positional information may be less sensitive to reconstruction than intensity, consistent with the robustness of these metrics to image filtering and voxel resampling reported in an independent NSCLC cohort [4].

Fourth, tumours were segmented by requiring each voxel to satisfy both the EANM-recommended isocontour of 41% of SUVmax and an absolute SUV threshold of 2.5. This combined rule was adopted because the oral tongue, unlike the low-uptake background of the lung, is surrounded by intrinsic muscle of variable physiological uptake and by metallic dental restorations that can cause attenuation-correction artefacts. The rule was conservative: in the least avid tumours, which were also the smallest, it delineated a smaller volume of interest than the isocontour alone would have. Reproducibility was indeed lowest in these small tumours. In the oral tongue, therefore, the spatial metrics may perform as imaging biomarkers within a more narrowly defined cohort than in the lungs or breasts, and the optimal thresholding strategy for organs with mixed physiological uptake warrants dedicated study.

## 5. Conclusions

In surgically treated OTC, higher NHOCmax was associated with shorter OS, and lower NHOPmax was associated with shorter DFS and OS upon univariable analysis, albeit without retaining significance after adjustment for stage and MTV. Both metrics correlated weakly with conventional PET parameters. In the reproducibility analysis, both were measured consistently in tumours larger than 2 cm^3^ across segmentation methods and on re-segmentation by the same reader, but each became less reliable in low-volume tumours. Both metrics therefore warrant evaluation in larger multicentre cohorts and in other head and neck subsites before their prognostic value can be established.

## Figures and Tables

**Table 1:** Baseline characteristics.

Characteristic	Total (*n* = 89)
Age (years), mean (SD)	52.1 (14.3)
Tumour size (cm), mean (SD)	2.6 (1.6)
Depth of invasion (cm), mean (SD)	1.1 (0.9)
Sex	
Male	52 (58%)
Female	37 (42%)
T stage	
T1	21 (24%)
T2	25 (28%)
T3	43 (48%)
N stage	
N0	54 (61%)
N1	13 (15%)
N2	14 (16%)
N3	8 (9.0%)
AJCC 8th stage	
I	21 (24%)
II	20 (22%)
III	26 (29%)
IV	22 (25%)
Differentiation	
Well	36 (40%)
Moderately	41 (46%)
Poorly	12 (13%)
Lymphovascular invasion	
Negative	64 (72%)
Positive	25 (28%)
Perineural invasion	
Negative	52 (58%)
Positive	37 (42%)
Surgical margin	
Negative	88 (99%)
Positive	1 (1.1%)
Adjuvant treatment	
Not done	59 (66%)
RT	14 (16%)
CCRT	15 (17%)
CT	1 (1.1%)

Abbreviations: RT = radiation; CCRT = chemoradiation; CT = chemotherapy; AJCC = American Joint Committee on Cancer.

**Table 2:** Distribution of PET parameters.

PET Parameter	Median [IQR]	Range (Min–Max)
SUVmax	7.98 [5.36, 11.77]	2.78–20.21
SUVmean	4.85 [3.44, 7.09]	2.62–13.58
MTV (cm^3^)	2.18 [1.25, 4.31]	0.23–65.15
TLG	13.13 [7.51, 25.03]	1.14–452.17
NHOCmax	0.39 [0.26, 0.60]	0.10–1.88
NHOPmax	0.43 [0.34, 0.50]	0.15–0.92

PET = positron emission tomography; IQR = inter-quartile range; SUVmax = maximum standardised uptake value; SUVmean = mean standardised uptake value; MTV = metabolic tumour volume; TLG = total lesion glycolysis; NHOCmax = normalised distance from the hotspot to the centroid; NHOPmax = normalised distance from the hotspot to the perimeter.

**Table 3:** Univariable analysis for DFS and OS.

Variable	DFS HR (95% CI)	*p*	OS HR (95% CI)	*p*
Age (per year)	1.02 (1.00–1.05)	0.093	1.03 (1.01–1.06)	0.014
Sex (male vs. female)	0.50 (0.22–1.12)	0.093	0.53 (0.22–1.28)	0.159
Tumour size (per cm)	1.35 (1.08–1.70)	0.009	1.49 (1.17–1.89)	0.001
Depth of invasion (per cm)	1.88 (1.32–2.68)	<0.001	2.39 (1.54–3.70)	<0.001
Lymphovascular invasion (positive vs. negative)	2.05 (0.96–4.38)	0.064	2.08 (0.93–4.64)	0.074
Perineural invasion (positive vs. negative)	2.34 (1.12–4.92)	0.025	3.08 (1.36–6.97)	0.007
AJCC stage (IV vs. I–III)	2.87 (1.37–6.02)	0.005	3.97 (1.81–8.74)	<0.001
Adjuvant treatment (any vs. none)	0.84 (0.38–1.85)	0.668	0.97 (0.42–2.24)	0.938
SUVmax (per unit)	1.00 (0.92–1.08)	0.985	1.01 (0.93–1.10)	0.821
SUVmean (per unit)	1.00 (0.87–1.14)	0.998	1.02 (0.89–1.17)	0.757
MTV (per cm^3^)	1.05 (1.02–1.08)	<0.001	1.09 (1.05–1.13)	<0.001
TLG (per unit)	1.01 (1.00–1.01)	<0.001	1.01 (1.01–1.02)	<0.001
NHOCmax (per 0.1)	1.11 (0.99–1.23)	0.063	1.14 (1.01–1.29)	0.032
NHOPmax (per 0.1)	0.65 (0.45–0.92)	0.017	0.65 (0.44–0.96)	0.031

Abbreviations: CI = confidence interval; HR = hazard ratio; SUVmax = maximum standardised uptake value; MTV = metabolic tumour volume; TLG = total lesion glycolysis; NHOCmax = normalised distance from the hotspot to the centroid; NHOPmax = normalised distance from the hotspot to the perimeter; AJCC = American Joint Committee on Cancer.

**Table 4:** Multivariable analysis for DFS and OS.

Model/Variable	DFS HR (95% CI)	DFS *p*	OS HR (95% CI)	OS *p*
Base model (AJCC stage + MTV)				
AJCC stage (IV vs. I–III)	2.21 (0.99–4.93)	0.054	2.75 (1.18–6.43)	0.020
MTV (per cm^3^)	1.04 (1.01–1.07)	0.014	1.07 (1.03–1.11)	<0.001
C-index	0.642 (0.531–0.750)		0.695 (0.569–0.816)	
Model 1: +NHOCmax				
AJCC stage (IV vs. I–III)	2.33 (1.05–5.17)	0.037	2.78 (1.20–6.41)	0.017
MTV (per cm^3^)	1.03 (1.00–1.07)	0.045	1.07 (1.03–1.11)	<0.001
NHOCmax (per 0.1)	1.08 (0.96–1.20)	0.199	1.09 (0.96–1.23)	0.182
C-index	0.656 (0.537–0.773)		0.703 (0.579–0.827)	
Model 2: +NHOPmax				
AJCC stage (IV vs. I–III)	2.00 (0.89–4.48)	0.092	2.48 (1.04–5.93)	0.040
MTV (per cm^3^)	1.03 (1.00–1.07)	0.046	1.07 (1.03–1.11)	<0.001
NHOPmax (per 0.1)	0.75 (0.53–1.06)	0.101	0.81 (0.56–1.18)	0.273
C-index	0.679 (0.571–0.781)		0.700 (0.585–0.813)	

Abbreviations: CI = confidence interval; HR = hazard ratio; AJCC = American Joint Committee on Cancer; MTV = metabolic tumour volume; NHOCmax = normalised distance from the hotspot to the centroid; NHOPmax = normalised distance from the hotspot to the perimeter.

**Table 5:** Intra-reader repeatability of the spatial metabolic metrics (NHOCmax and NHOPmax) between an original and a repeated manual segmentation by a single reader (*n* = 30), expressed as ICC(2,1) with 95% confidence intervals overall and by reference MTV stratum.

Metric	Overall	<1 (*n* = 8)	1–2 (*n* = 8)	2–5 (*n* = 7)	≥5 (*n* = 7)
NHOCmax	0.69 (0.44 to 0.84)	0.15 (−0.51 to 0.73)	0.52 (−0.32 to 0.88)	0.85 (0.34 to 0.97)	0.92 (0.66 to 0.99)
NHOPmax	0.94 (0.89 to 0.97)	0.97 (0.86 to 0.99)	0.87 (0.48 to 0.97)	0.97 (0.85 to 1.00)	0.99 (0.93 to 1.00)

Abbreviations: ICC = intra-class correlation coefficient; CI = confidence interval; MTV = metabolic tumour volume; NHOCmax = normalised distance from the hotspot to the centroid; NHOPmax = normalised distance from the hotspot to the perimeter.

**Table 6:** Reproducibility of the spatial metabolic metrics (NHOCmax and NHOPmax) across segmentation methods, expressed as ICC(2,1) with 95% confidence intervals overall and by reference MTV stratum (*n* = 88; six threshold-based segmentations).

Metric	Overall	<1 (*n* = 17)	1–2 (*n* = 20)	2–5 (*n* = 32)	≥5 (*n* = 19)
NHOCmax	0.94 (0.92 to 0.96)	0.91 (0.83 to 0.96)	0.89 (0.80 to 0.95)	0.97 (0.95 to 0.98)	0.95 (0.89 to 0.98)
NHOPmax	0.78 (0.71 to 0.83)	0.55 (0.35 to 0.76)	0.76 (0.61 to 0.88)	0.73 (0.61 to 0.84)	0.83 (0.71 to 0.92)

Abbreviations: ICC = intra-class correlation coefficient; CI = confidence interval; MTV = metabolic tumour volume; NHOCmax = normalised distance from the hotspot to the centroid; NHOPmax = normalised distance from the hotspot to the perimeter.

## Data Availability

The spatial PET metrics (NHOCmax and NHOPmax) were computed within LIFEx (version 25.06.1). The analysis code and the in-house segmentation pipeline used for the robustness analyses are available from the corresponding author upon reasonable request. Individual patient data cannot be shared owing to institutional and patient-privacy restrictions.

## Supplementary Materials

The following supporting information can be downloaded at: https://www.mdpi.com/article/10.3390/cancers18162637/s1. Figure S1: Kaplan–Meier curves for NHOCmax (first column) and NHOPmax (second column), each dichotomised at its median value, with disease-free survival in the top row and overall survival in the bottom row. The median cutoff for each metric is indicated in the panel legend (low: ≤cutoff; high: >cutoff), the log-rank *p*-value is shown within each panel, and the numbers at risk are tabulated below each curve. Figure S2: Kaplan–Meier curves for the conventional PET parameters (SUVmax, MTV, and TLG), each dichotomised at its median value, with disease-free survival in the left column and overall survival in the right column. The median cutoff for each metric is indicated in the panel legend (low: ≤cutoff; high: >cutoff), the log-rank *p*-value is shown within each panel, and the numbers at risk are tabulated below each curve. Table S1: PET/CT scanners, acquisition periods, reconstruction matrix, voxel size, and reconstruction algorithms in the study cohort (*n* = 89). Voxel size is given as in-plane × in-plane × slice thickness; values were uniform within each scanner group. Table S2: Reproducibility of the SUVpeak-based spatial metrics (NHOCpeak and NHOPpeak) across the six threshold-based segmentations, expressed as ICC(2,1) (two-way random-effects, single-measure, absolute agreement) with 95% confidence intervals. Because SUVpeak is averaged over a fixed 1-cm^3^ sphere, the peak metrics could be computed in all six segmentations for only 22 tumours, predominantly larger lesions; volume strata below 2 cm^3^ contained too few tumours (*n* ≤ 1) for stratified estimation (shown as –). The number of tumours per stratum is given in the bottom row. The corresponding maximum-voxel metrics (NHOCmax and NHOPmax) are given in Table 6. Table S3: Sensitivity analysis of the spatial metrics (NHOCmax and NHOPmax) restricted to tumours with MTV ≥ 1 cm^3^ (*n* = 71). Each spatial index was entered separately; multivariable models were adjusted for AJCC stage and MTV, and the incremental value over stage and MTV was assessed by nested likelihood-ratio tests. Hazard ratios are per 0.1-unit increment, reported with 95% confidence intervals, for disease-free survival (DFS) and overall survival (OS).

## Abbreviations

The following abbreviations are used in this manuscript:

OTC	oral tongue cancer
FDG	fluorodeoxyglucose
PET/CT	positron emission tomography/computed tomography
SUVmax	maximum standardised uptake value
SUVmean	mean standardised uptake value
MTV	metabolic tumour volume
TLG	total lesion glycolysis
NHOCmax	normalised distance from the hotspot to the centroid
NHOPmax	normalised distance from the hotspot to the perimeter
DFS	disease-free survival
OS	overall survival
AJCC	American Joint Committee on Cancer
ICC	Intra-class correlation coefficient
LVI	lymphovascular invasion
PNI	perineural invasion
DOI	depth of invasion
IQR	interquartile range
DICOM	Digital Imaging and Communications in Medicine
OSEM	ordered-subset expectation maximisation
PSF	point-spread function
TOF	time-of-flight
