# Supplementary material for: Reproducibility and Potential Prognostic Value of Spatial Metabolic PET Metrics in Oral Tongue Squamous Cell Carcinoma
Source: Cancers (Basel). 2026 Aug 15;18(16):2637. doi: 10.3390/cancers18162637 (PMC13510975; doi:10.3390/cancers18162637)
Supplement: Supplementary file 1 [file cancers-18-02637-s001.zip › cancers-4457810-supplementary.pdf]

Supplementary Materials

Spatial metabolic PET metrics (NHOCmax and NHOPmax) in surgically treated oral tongue cancer

---

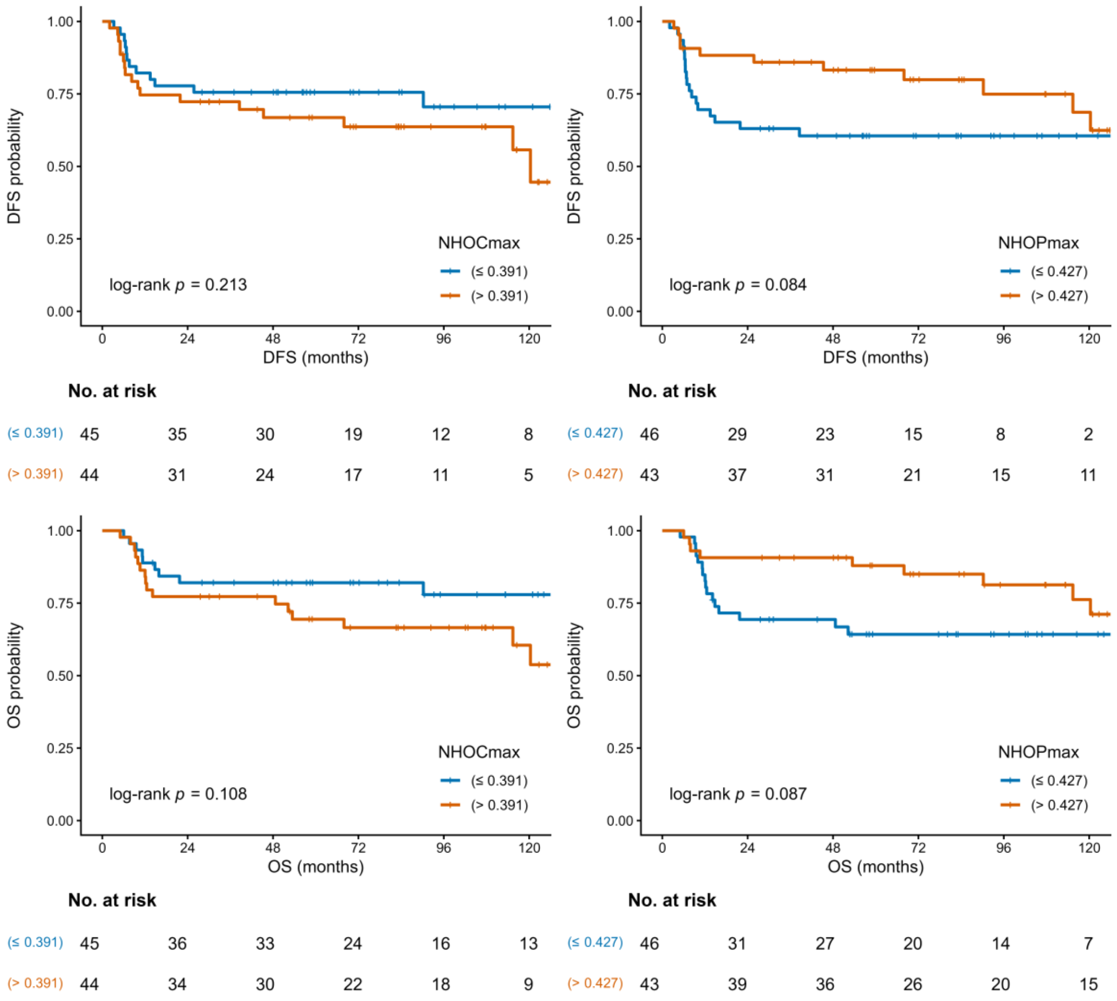

Supplementary Figure S1. Kaplan–Meier curves for NHOCmax (first column) and NHOPmax (second column), each dichotomised at its median value, with disease-free survival in the top row and overall survival in the bottom row. The median cutoff for each metric is indicated in the panel legend (low: ≤ cutoff; high: > cutoff), the log-rank *p*-value is shown within each panel, and the numbers at risk are tabulated below each curve.

Abbreviations: DFS = disease-free survival; OS = overall survival; NHOCmax = normalised distance from the hotspot to the centroid; NHOPmax = normalised distance from the hotspot to the perimeter.

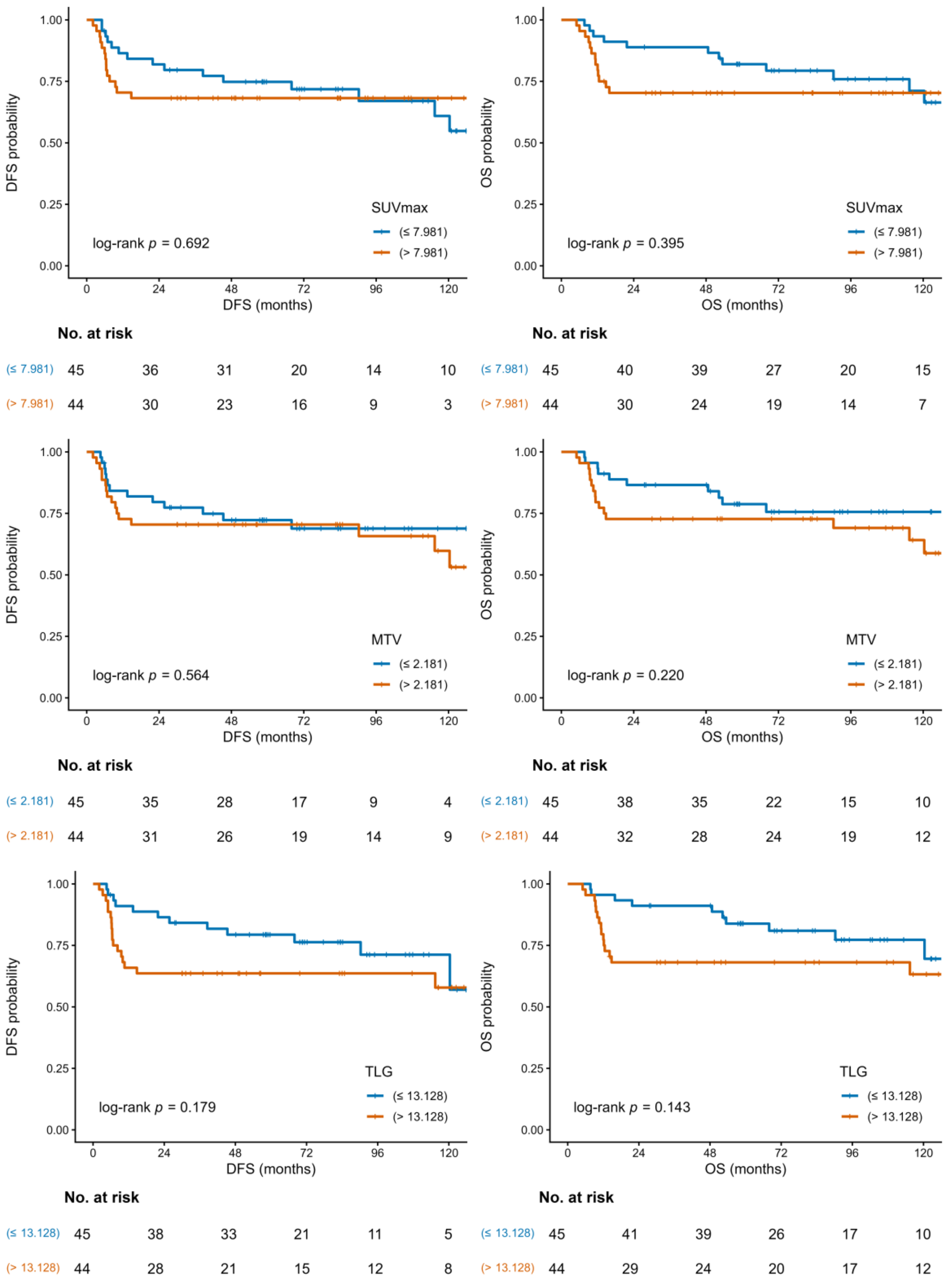

Supplementary Figure S2. Kaplan–Meier curves for the conventional PET parameters (SUVmax, MTV, and TLG), each dichotomised at its median value, with disease-free survival

in the left column and overall survival in the right column. The median cutoff for each metric is indicated in the panel legend (low: ≤ cutoff; high: > cutoff), the log-rank *p*-value is shown within each panel, and the numbers at risk are tabulated below each curve.

Abbreviations: DFS = disease-free survival; OS = overall survival; SUVmax = maximum standardised uptake value; MTV = metabolic tumour volume; TLG = total lesion glycolysis.

Supplementary Table S1. PET/CT scanners, acquisition periods, reconstruction matrix, voxel size, and reconstruction algorithms in the study cohort (*n* = 89). Voxel size is given as in-plane × in-plane × slice thickness; values were uniform within each scanner group.

Scanner	Acquisition period	n	Matrix	Voxel size (mm)	Reconstruction
Biograph DUO (Siemens)	2003–2012	16	128 × 128	5.31 × 5.31 × 3.38	OSEM (2 iter, 8 subsets)
Biograph TruePoint (Siemens)	2007–2023	46	168 × 168	4.07 × 4.07 × 5.00	OSEM (4 iter, 8 subsets) (n = 29); OSEM + PSF (3 iter, 21 subsets) (n = 17)
Discovery 710 (GE)	2014–2023	27	192 × 192	3.65 × 3.65 × 3.27	VPFXS (TOF + PSF)

Abbreviations: OSEM = ordered-subset expectation maximisation; PSF = point-spread function; TOF = time-of-flight; VPFXS = VUE Point FX with SharpIR.

Supplementary Table S2. Reproducibility of the peak-based spatial metrics (NHOCpeak and NHOPpeak) across the six threshold-based segmentations, expressed as ICC(2,1) (two-way random-effects, single-measure, absolute agreement) with 95% confidence intervals. Because SUVpeak is averaged over a fixed 1-cm^3 sphere, the peak metrics could be computed in all six segmentations for only 22 tumours, predominantly larger lesions; volume strata below 2 cm^3 contained too few tumours (n ≤ 1) for stratified estimation (shown as –). The number of tumours per stratum is given in the bottom row. The corresponding maximum-voxel metrics (NHOCmax and NHOPmax) are given in Table 6.

Metric	Overall	<1	1–2	2–5	≥5
NHOCpeak	0.93 (0.88 to 0.97)	–	–	0.87 (0.61 to 0.99)	0.93 (0.87 to 0.97)
NHOPpeak	0.88 (0.80 to 0.94)	–	–	0.51 (0.15 to 0.94)	0.90 (0.81 to 0.95)
n	22	0	1	4	17

Reference metabolic tumour volume strata are in cm^3. Abbreviations: ICC = intraclass correlation coefficient; CI = confidence interval; NHOCpeak/NHOPpeak = peak-based normalised distances from the hotspot to the centroid/perimeter.

Supplementary Table S3. Sensitivity analysis of the spatial metrics (NHOCmax and NHOPmax) restricted to tumours with MTV ≥ 1 cm^3 (n = 71). Each spatial index was entered separately; multivariable models were adjusted for AJCC stage and MTV, and the incremental value over stage and MTV was assessed by nested likelihood-ratio tests. Hazard ratios are per 0.1-unit increment, reported with 95% confidence intervals, for disease-free survival (DFS) and overall survival (OS).

Analysis	Metric	DFS HR (95% CI)	DFS *p*	OS HR (95% CI)	OS *p*
Univariable	NHOCmax	1.11 (1.00–1.23)	0.05	1.15 (1.02–1.30)	0.03
Univariable	NHOPmax	0.67 (0.45–1.00)	0.05	0.68 (0.44–1.04)	0.08
Multivariable	NHOCmax	1.08 (0.97–1.22)	0.16	1.10 (0.97–1.25)	0.15
Multivariable	NHOPmax	0.76 (0.52–1.13)	0.17	0.82 (0.54–1.24)	0.34

Multivariable models were adjusted for AJCC stage (IV vs I–III) and MTV (per cm^3); NHOCmax and NHOPmax were entered in separate models. Nested likelihood-ratio test for adding the spatial index to AJCC stage + MTV: DFS + NHOCmax p = 0.20; DFS + NHOPmax p = 0.16; OS + NHOCmax p = 0.19; OS + NHOPmax p = 0.33. Abbreviations: HR = hazard ratio; CI = confidence interval; MTV = metabolic tumour volume; AJCC = American Joint Committee on Cancer.
